# In Vitro Effect of Pitavastatin and Its Synergistic Activity with Isavuconazole against *Acanthamoeba castellanii*

**DOI:** 10.3390/pathogens9090681

**Published:** 2020-08-21

**Authors:** Hye Jee Hahn, Jose Ignacio Escrig, Brian Shing, Anjan Debnath

**Affiliations:** Center for Discovery and Innovation in Parasitic Diseases, Skaggs School of Pharmacy and Pharmaceutical Sciences, University of California, San Diego, CA 92093, USA; lilyh814@gmail.com (H.J.H.); nachoeslar@hotmail.com (J.I.E.); bjshing@health.ucsd.edu (B.S.)

**Keywords:** *Acanthamoeba*, free-living ameba, *Acanthamoeba* keratitis, statin, CYP51 inhibitor

## Abstract

*Acanthamoeba* keratitis (AK) can occur in healthy individuals wearing contact lenses and it is a painful, blinding infection of the cornea caused by a free-living ameba *Acanthamoeba*. Current treatment for AK relies on a combination of chlorhexidine, propamidine isethionate, and polyhexamethylene biguanide. However, the current regimen includes an aggressive disinfectant and in 10% of cases recurrent infection ensues. Therefore, development of efficient and safe drugs is a critical unmet need to avert blindness. *Acanthamoeba* sterol biosynthesis includes two essential enzymes HMG-CoA reductase (HMGR) and sterol 14-demethylase (CYP51), and we earlier identified a CYP51 inhibitor isavuconazole that demonstrated nanomolar potency against *A. castellanii* trophozoites. In this study, we investigated the effect of well-tolerated HMGR inhibitors and identified pitavastatin that is active against trophozoites of three different clinical strains of *A.* *castellanii*. Pitavastatin demonstrated an EC_50_ of 0.5 to 1.9 µM, depending on strains. Combination of pitavastatin and isavuconazole is synergistic and led to 2- to 9-fold dose reduction for pitavastatin and 11- to 4000-fold dose reduction for isavuconazole to achieve 97% of growth inhibition. Pitavastatin, either alone or in combination with isavuconazole, may lead to repurposing for the treatment of *Acanthamoeba* keratitis.

## 1. Introduction

Painful blinding keratitis is caused by the free-living ameba *Acanthamoeba* and can occur in healthy individuals wearing contact lenses [1]. *Acanthamoeba* keratitis (AK) is a rare but serious infection of the eye that causes inflammation in the clear front surface of the eye (the cornea) and can result in permanent visual impairment or blindness. *Acanthamoeba* is common in nature and can be found in soil, air and water, including insufficiently chlorinated pools, hot tubs, tap and shower water. In unfavorable environments, the ameboid form of the organism called a ‘trophozoite’ transforms into a drug-resistant double-walled cyst. Cyst resistance to therapeutic agents, and recurrence of infection due to *Acanthamoeba* excystment, remain a challenge for disease prevention and cure.

AK is most common in people who wear contact lenses, but anyone can develop the infection [2,3,4]. The incidence of the disease in the United States has been conservatively estimated at approximately one to two cases per million contact lens users [5]. However, it is possible that the infection is underdiagnosed, as a retrospective study from a single center in Iowa showed that the average number of new AK cases per year among Iowa residents doubled during 2010–2017 [6]. About 85% of American patients affected with *Acanthamoeba* are contact lens wearers [1]. In other countries such as India, *Acanthamoeba* infections are widely reported in non-contact lens wearers [7]. Infection recurrence due to *Acanthamoeba* excystment occurs in approximately 10% of cases. Complications of AK include dacryoadenitis, corneal melting and scarring, severe secondary glaucoma, and chronic anterior segment inflammation [8]. Scleral inflammation, often referred to as sclerokeratitis, may also develop [9]. Because of the rapid increase in the case number, AK has been listed by the National Institutes of Health as an Emerging Infectious Disease.

Current treatment of AK involves an aggressive disinfectant chlorhexidine, in combination with diamidines, polyhexamethylene biguanide, (PHMB) and neomycin, and can last up to a year. Combination therapies have proven more successful than single-drug therapies [10,11,12]. Corticosteroids are applied topically to control corneal inflammation, pain, and scleritis, particularly following keratoplasty [13]. Despite advances in combination therapies and surgery, recurrence of infection remains a challenge that is yet to be addressed [14]. Therefore, the development of efficient drugs is a critical unmet need to avert blindness.

Since ergosterol is one of the major sterols in the membrane of free-living amebae [15,16,17,18] and both trophozoites and cysts of *Acanthamoeba* require sterols [18], disruption of isoprenoid and sterol biosynthesis by small-molecule inhibitors may be an effective intervention strategy against AK. To perform ergosterol synthesis, *A. castellanii*, among other enzymes, encodes for a CYP51 enzyme, which has ~31–35% sequence identity to fungal CYP51 [19]. The genome of *A. castellanii* also contains gene encoding 3-hydroxy-3-methylglutaryl-coenzyme A (HMG-CoA) reductase (HMGR), which catalyzes the conversion of HMG-CoA to mevalonate, one of the early precursors for the production of isoprenoids and subsequently ergosterol. Earlier, we identified isavuconazole as the most potent CYP51 inhibitor tested against *A. castellanii* trophozoites and isavuconazole suppressed excystment of preformed *Acanthamoeba* cysts into trophozoites [20]. In this study, we tested the effect of different HMGR inhibitors, also known as statins, against trophozoites of three clinical strains of *A. castellanii* and investigated the effect of the combination of the two most potent inhibitors of two essential enzymes in the *Acanthamoeba* sterol biosynthesis, HMGR and CYP51.

## 2. Results and Discussion

### 2.1. In Vitro Activity of HMGR Inhibitors against Clinical Strains of A. castellanii

HMGR catalyzes the conversion of HMG-CoA into mevalonate [21]. HMGR inhibitors developed as cholesterol lowering drugs, also known as statins, prevent the conversion of HMG-CoA to mevalonate, resulting in the inhibition of the isoprenoid biosynthesis and the downstream sterol biosynthesis [22]. Although inhibition of *Acanthamoeba* HMGR and parasite growth was evaluated elsewhere using siRNA and statins [23,24], none of the previously tested statins [23,24] showed potency against *A. castellanii* comparable to chlorhexidine, a disinfectant currently used for the treatment of AK. Amebicidal effect demonstrated earlier [23,24] by selected statins encouraged us to systematically assess this drug class for inhibition of *A. castellanii*.

We tested six statins, fluvastatin, atorvastatin, simvastatin, rosuvastatin, pravastatin and pitavastatin, against *A. castellanii* trophozoites of Ma strain. While fluvastatin, atorvastatin and rosuvastatin exhibited similar EC_50_, ranging from 10 to 12.5 µM, we demonstrated that the HMGR inhibitor pitavastatin (marketed as LIVALO^TM^) was equipotent (EC_50_ = 1.9 µM) to chlorhexidine and superior to PHMB and all previously tested statins. We also tested pitavastatin against two other clinical strains of T4 genotype, CDC:V240 and MEEI 0184, and found that both strains were equally susceptible to pitavastatin (EC_50_ of 1 µM and 0.5 µM) (Table 1). Fluvastatin, atorvastatin, simvastatin and pravastatin were tested earlier against *A. castellanii* T4 genotypes [24]. Although a different assay method, 96-h time point and different clinical strains were used by Martin-Navarro et al. [24] to determine the EC_50_ of these four statins, the activity of fluvastatin, atorvastatin and pravastatin reported earlier [24] was comparable to the EC_50_ obtained in our study. Our study, on the other hand, identified pitavastatin as the most potent statin against *A. castellanii* and is favorably comparable to the current standard of care chlorhexidine.

Since pitavastatin is an FDA-approved drug, the safety data of pitavastatin are widely available. No apparent cytotoxicity was observed when pitavastatin was tested at 10 µM against human T lymphocytes [25]. It demonstrated an EC_50_ of about 20 µM in HepG2 and HEK293 cells. This provides a selectivity index of 10–40, depending on cell types and on the strains of *A. castellanii* [26]. Clinically, there may be some concerns about the reported cataractogenic effect of statins. According to the LIVALO^®^ (Pitavastatin) label, cataracts and lens opacities were seen in dogs treated for 52 weeks. Binding studies of pitavastatin using lens protein of various species (including humans) showed that the dog lens had a higher level of binding than other species and therefore it is less likely that humans are at risk of developing cataracts. Furthermore, recent analysis of correlations between statin use and cataracts did not find evidence that statins increase the risk of cataracts [27]. Finally, considering a shorter treatment schedule for AK, we would expect the minimum adverse effects of pitavastatin on lens transparency.

### 2.2. Effect of Combination of Pitavastatin and Isavuconazole

Blocking two essential enzymes in the sterol pathway is more detrimental for unicellular organisms than inhibiting one [17,28]. In a recent study, we identified isavuconazole with nanomolar potency against different *A. castellanii* clinical strains of the T4 genotype [20]. In this study, we determined the synergistic effect of pitavastatin (HMGR inhibitor) and isavuconazole (CYP51 inhibitor) at fixed concentration ratios. The dose–effect relationships between two drugs were assessed by classical isobolograms built to calculate Chou-Talalay combination indices and dose-reduction indices, using the CompuSyn software [29]. The calculated parameters indicated synergy (CI values from 0.2 to 0.7) at different drug ratios. Thus, 97% growth inhibition with 2- to 9-fold dose reduction for pitavastatin and 11- to 4000-fold dose reduction for isavuconazole was achieved at 4:1, 8:1, 16:1, 32:1, 2000:1, and 4000:1 ratio of pitavastatin and isavuconazole (Table 2). FDA-approved isavuconazole (marketed as a prodrug Cresemba^TM^) has a good safety profile and is well-tolerated in humans [30]. Isavuconazole CC_50_ against human A549 lung carcinoma cells was found > 10 µM [31]. This provides a selectivity index of about 400 to 10,000, depending on the strains of *A. castellanii*. Since the concentrations of pitavastatin and isavuconazole required to show synergism are lower than the concentrations that exhibit toxicity against mammalian cells, we believe that the concentrations used in the combination study will not produce significant toxicity on mammalian cells.

Although topical administrations in human are not currently available for pitavastatin and isavuconazole, pitavastatin is rapidly absorbed after oral administration and reaches peak plasma concentrations in human within 1 hr. The elimination half-life of pitavastatin is approximately 12 h [32] and it is mainly distributed in the liver [33]. Maximum plasma levels of isavuconazole were detected 2–3 h after oral administration and it has a prolonged half-life (100–130 h) [34,35,36]. Isavuconazole is widely distributed in liver, lungs, eyes, kidneys, bone, nasal mucosa, and brain [30]. Statins have been used in a wide range of eye disorders like indistinct soft drusen [37] and vasodilation in retinal venules and arterioles [38]. Precedents of topical administration of statins in mouse models include simvastatin for host protection against cutaneous leishmaniasis caused by *Leishmania major* [39] and as an antibacterial agent against methicillin-resistant *Staphylococcus aureus* infection [40]. Formulation for aqueous and oil suspension and preparations for topical and local application of statins are available [41]. Ophthalmic formulation has also been developed to topically administer econazole having poor aqueous solubility [42]. These studies lend support to the potential use of HMGR and CYP51 inhibitors as topical agents in the treatment of AK.

### 2.3. Microscopy Study to Determine the Effect of Combination of Pitavastatin and Isavuconazole

**Figure 1:**
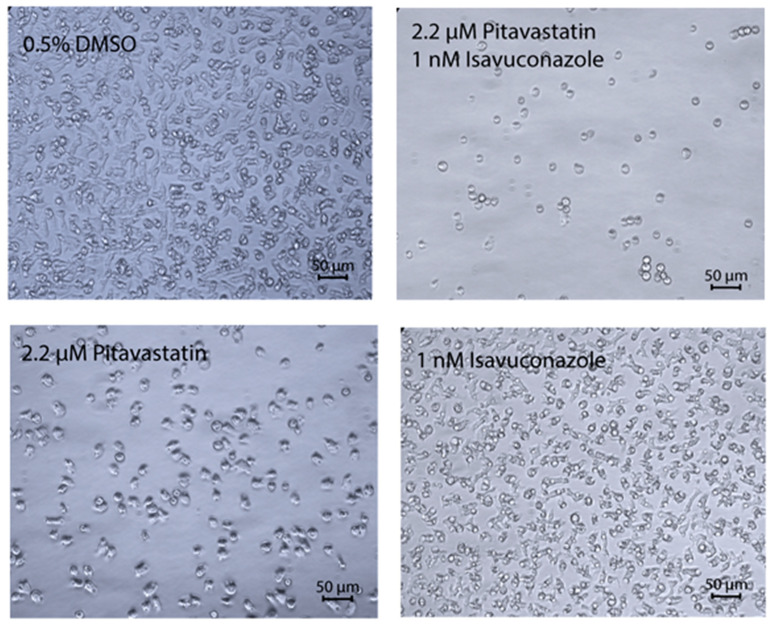
Synergistic effect of pitavastatin and isavuconazole. The phase contrast microscope images show *A. castellanii* Ma trophozoites treated for 48 h with 0.5% DMSO and a combination of 2.2 μM of pitavastatin and 0.001 µM of isavuconazole. The drug-treated *A. castellanii* cells are rounded, and much smaller in size, whereas DMSO-treated cells are irregularly shaped with visible cytoplasm. Magnification, ×20.

The combination of pitavastatin and isavuconazole that generated the highest synergy was further analyzed by microscopy study to understand the effect of both these drugs on the growth and morphology of *A. castellanii*. The pitavastatin-isavuconazole pair, combined at concentrations of 2.2 and 0.001 µM, respectively, produced a CI value of 0.2 (Table 2). When combined at this ratio, these two drugs caused almost no growth of cells at 48 h, cells were rounded and much smaller in size. Trophozoites treated with the same concentration of pitavastatin alone or isavuconazole alone grew normally, albeit at a slower rate than 0.5% DMSO-treated control cells which appeared normal (Figure 1).

Statins and azoles are known to induce apoptosis in different mammalian cells [43,44]. An earlier study also confirmed that atorvastatin, fluvastatin, simvastatin and voriconazole induced programmed cell death (PCD) in *A. castellanii* [23]. Whether pitavastatin or isavuconazole, either alone or in combination, leads to PCD in *A. castellanii*, requires further investigation.

## 3. Materials and Methods

### 3.1. In Vitro Activity of HMGR Inhibitors against Clinical Strains of A. castellanii

The trophozoites of the *A. castellanii* Ma strain (ATCC 50370), CDC:V240 strain (acquired from CDC, Atlanta, GA, USA) and MEEI 0184 (acquired from Tufts University, Boston, MA, USA), belonging to T4 genotype [45], were cultured axenically in PYG medium supplemented with 1% penicillin-streptomycin at 28 °C [20]. All experiments were performed with trophozoites harvested during the logarithmic phase of growth.

A primary screen with HMGR inhibitors was performed in triplicate in three independent biological replicates, at a single drug concentration, 50 µM, against *A. castellanii* Ma trophozoites (5000 cells/well). For this experiment in a 96-well microtiter plate, 0.5 µL of the 10 mM stock solution of fluvastatin, atorvastatin, simvastatin, rosuvastatin, pitavastatin, and pravastatin (MilliporeSigma, St. Louis, MO, USA; Tocris, Minneapolis, MN, USA; LC Laboratories, Woburn, MA, USA), dissolved in DMSO was added into each well to yield a final concentration of 50 µM in 0.5% DMSO. For a negative control, 0.5% DMSO (MilliporeSigma, St. Louis, MO, USA) was used and 50 µM of chlorhexidine (Fisher Scientific, Waltham, MA, USA) was used as a positive control. The statins were incubated for 48 h in the presence of 5000 trophozoites in each well. The activity of each statin on trophozoites was determined by measuring the ATP bioluminescence using CellTiter-Glo luminescence-based viability assay (Promega, Madison, WI, USA) [46]. Hits showing ≥ 80% inhibition were further tested in triplicate in three independent experiments (biological replicates) to determine EC_50_ values.

For EC_50_ determination in a 96-well plate, 20 mM stock of simvastatin and 10 mM stocks of fluvastatin, atorvastatin, rosuvastatin, and pitavastatin were first serially diluted in a clear bottom 96-well working plate to yield a concentration range of 20 to 0.156 mM for simvastatin and 10 to 0.078 mM for other statins. 0.5 µL of each concentration of the compound in the working plate was then transferred in triplicate to the respective row of the 96-well screening plate. A total of 99.5 µL of *A. castellanii* Ma trophozoites (5000 cells) were added to the screening plate to yield final concentrations ranging from 100 to 0.78 µM for simvastatin and 50 to 0.39 µM for other statins in 0.5% DMSO. Negative and positive control wells in the screening plates contained 0.5% DMSO and 50 µM chlorhexidine, respectively. The dose response of pitavastatin was also determined against CDC:V240 and MEEI 0184 strains. After incubation of assay plates for 48 h at 28 °C, 25 µL of CellTiter-Glo luminescent cell viability assay reagent (Promega, Madison, WI, USA) was added to each well of the 96-well plate. Cell lysis was induced by shaking the plate at room temperature for 10 min. The plate was then kept in the dark at room temperature for 5 min and the resulting ATP bioluminescence of the trophozoites was measured by an EnVision plate reader (PerkinElmer, Waltham, MA, USA) [20]. All experiments were conducted in triplicate. Statistical analysis of experiments and determination of EC_50_ values was performed by using GraphPad Prism software 5.0 (GraphPad, San Diego, CA, USA).

### 3.2. Effect of Combination of Pitavastatin and Isavuconazole

We earlier showed that CYP51 inhibitor isavuconazole elicited potent activity against trophozoites of different strains of *A. castellanii* [20]. Since both isavuconazole and pitavastatin inhibit enzymes in the ergosterol biosynthetic pathway, we tested the activity of the combination of pitavastatin and isavuconazole against *A. castellanii* Ma trophozoites and compared the effect of the combination of these two drugs with the effect of a single drug. The growth inhibition was determined by CellTiter-Glo assay (Promega, Madison, WI, USA). Briefly, 0.25 µL from 10 mM stock of pitavastatin and 0.25 µL from 80 µM stock of isavuconazole were transferred to the combination well of the 96-well assay plate. This provided a final concentration of 25 µM for pitavastatin and 0.2 µM for isavuconazole that induced 100% growth inhibition. Similarly, 0.25 µL each of serially diluted pitavastatin and isavuconazole was transferred in each well of the 96-well assay plate in a checkerboard fashion. The plate with different ratios of pitavastatin and isavuconazole also contained a column of pitavastatin alone, ranging from 25 to 0.195 µM, and a column of isavuconazole alone, ranging from 0.2 to 0.00156 µM, to determine the EC_50_ of individual drugs. Each plate contained 0.5% DMSO as a negative control and 50 µM of chlorhexidine as a positive control. A total of 5000 *A. castellanii* Ma trophozoites in 99.5 µL of PYG medium added in each well of the 96-well plate and the trophozoites were incubated for 48 h at 28 °C. The growth inhibition of trophozoites in the presence of pitavastatin and isavuconazole, both alone and in combination, was measured by CellTiter-Glo ATP bioluminescence assay (Promega, Madison, WI, USA). All experiments were performed in triplicate. The effect of the combination of two drugs was calculated by the Chou-Talalay Combination Index method using CompuSyn software [29].

### 3.3. Microscopy Study to Determine the Effect of Combination of Pitavastatin and Isavuconazole

To confirm the synergistic effect of pitavastatin and isavuconazole, as predicted by the CompuSyn software, 5000 *A. castellanii* Ma trophozoites in PYG medium were incubated with 2.2 µM of pitavastatin alone, 0.001 µM of isavuconazole alone, and a combination of 2.2 µM of pitavastatin and 0.001 µM of isavuconazole in a 96-well clear bottom plate at 28 °C. Since a 4000:1 ratio of pitavastatin and isavuconazole provided the best combination index in CompuSyn to achieve 97% growth inhibition, we selected the pitavastatin-isavuconazole pair at this ratio in the microscopy study. Each plate contained trophozoites treated with 0.5% DMSO as a negative control. The effect of pitavastatin and isavuconazole, both alone and in combination, on trophozoites was imaged at 48 h under Zeiss Axiovert 40 CFL phase contrast microscope (Carl Zeiss, White Plains, NY, USA).

## 4. Conclusions

Our study identified a potent HMGR inhibitor pitavastatin that inhibited growth of trophozoites of different clinical strains of *A. castellanii* representing the T4 genotype. Combination of pitavastatin and isavuconazole led to a synergistic effect and allowed us to reduce concentrations of both drugs by an order of magnitude to achieve 97% of growth inhibition. Since both HMGR and CYP51 are ‘druggable’ targets in *A. castellanii* [16,24] and isavuconazole suppressed excystment of *Acanthamoeba* cysts into trophozoites [20], it is possible that combination of pitavastatin and isavuconazole at lower concentrations may also prevent recurrence of infection caused by *Acanthamoeba* excystment. Future studies will confirm the target of pitavastatin in *A. castellanii* and will investigate its effect on cysts, either alone or in combination with isavuconazole.

## Figures and Tables

**Table 1:** EC_50_ values of HMG-CoA reductase (HMGR) inhibitors against trophozoites of *A. castellanii.*

HMGR Inhibitors	Strain	EC_50_ (µM) Mean ± SE
Fluvastatin	Ma	11 ± 0.04
Atorvastatin	Ma	9.6 ± 0.04
Simvastatin	Ma	52.8 ± 0.2
Rosuvastatin	Ma	12.5 ± 2.3
Pravastatin	Ma	Not active
Pitavastatin	Ma	1.9 ± 0.03
	CDC:V240	1 ± 0.03
	MEEI 0184	0.5 ± 0.01
**Standards of Care**		
Chlorhexidine [20]	Ma	2 ± 0.07
	CDC:V240	1.1 ± 0.1
	MEEI 0184	1 ± 0.05
PHMB [20]	Ma	7.2 ± 0.06
	CDC:V240	11.8 ± 0.02
	MEEI 0184	4.6 ± 0.03

**Table 2:** Summary of synergism assay with pitavastatin and isavuconazole, shown for 97% growth inhibition of *A. castellanii* trophozoites.

Pitavastatin: Isavuconazole Ratio	% Growth Inhibition	Combination Index (CI)	Dose Reduction Index (DRI)		Dose Required to Achieve 97% Inhibition (µM)	
			Pitavastatin	Isavuconazole	Pitavastatin	Isavuconazole
4000:1	97	0.2 ± 0.1	4.3 ± 0.2	4077.8 ± 2.1	2.2 ± 0.1	0.0005 ± 0.0001
2000:1	97	0.5 ± 0.1	2.6 ± 0.6	1630.6 ± 114	4.2 ± 0.4	0.002 ± 0.0001
32:1	97	0.7 ± 0.1	2.0 ± 0.5	20.0 ± 12.0	5.4 ± 0.6	0.2 ± 0.04
16:1	97	0.5 ± 0.1	2.8 ± 0.2	12.8 ± 3.8	3.3 ± 0.4	0.2 ± 0.01
8:1	97	0.4 ± 0.1	4.8 ± 0.5	11.5 ± 4.5	2.0 ± 0.4	0.3 ± 0.06
4:1	97	0.3 ± 0.1	9.2 ± 1.3	11.1 ± 5.6	1.1 ± 0.1	0.3 ± 0.02
